# A Case of Primary Extragonadal Germ Cell Malignancy With Testicular Microlithiasis: Its Association and Significance

**DOI:** 10.7759/cureus.80018

**Published:** 2025-03-04

**Authors:** Anirudh Sreenivas, Suresh Kumar, Bhargavi M V, Srinivasan Ramadurai, Rajkumar Mani

**Affiliations:** 1 General Medicine, Sri Ramachandra Institute of Higher Education and Research, Chennai, IND

**Keywords:** extragonadal germ cell tumors, germ cell tumours (gct), mediastinal germ cell tumor, mediastinal seminoma, testicular microlithiasis

## Abstract

A 30-year-old South Asian male with no specific medical history presented with shortness of breath of insidious onset for two months, with facial puffiness and progressive dysphagia. Physical examination revealed dilated veins over the upper chest, upper arms, and back, with an ejection systolic murmur. Thoracic imaging revealed a large mass occupying the anterior mediastinum, encasing the aorta with mass effect on the esophagus, and causing superior vena cava obstruction. Histopathological and immunohistochemical examination of the mass revealed a seminomatous germ cell tumor. Ultrasonography of the testes was normal except for microlithiasis. The patient was initiated on cisplatin-based chemotherapy and showed symptomatic improvement after two cycles. This case describes an uncommon presentation of primary extragonadal germ cell malignancy with testicular microlithiasis, a premalignant condition linked to gonadal germ cell malignancy. Further studies are needed to determine a correlation, if any, between testicular microlithiasis and extragonadal germ cell malignancy.

## Introduction

Germ cell tumors (GCTs) of the testes constitute approximately 1-2% of all tumors in males aged 15-35 years. The overall incidence of all GCTs is 56.3 per million people, based on various registry-based case series [1]. Testicular microlithiasis (TM) is characteristically recognized by a sonographic pattern of multiple 1-3 mm hyperechoic foci distributed randomly throughout the testicular parenchyma. TM can be an incidental finding in approximately 5% of asymptomatic young men [2]. Patients with microlithiasis are at a 12-fold higher risk of developing testicular cancer than individuals without microlithiasis [3].

Extragonadal germ cell tumors (EGGCTs) are a type of germ cell neoplasm found outside the gonads without a gonadal primary. They may often be found in the mediastinum, retroperitoneum, or coccyx, at a rate of only 5-10% of all germ cell malignancies [4]. In the case of a primary EGGCT, the testicular ultrasound will be negative, or on rare occasions, it may show microlithiasis. Less than 10 cases of a primary extragonadal germ cell malignancy associated with TM have been reported worldwide thus far. Further studies are needed to study the association between TM and EGGCTs, which may provide insights into whether TM may hold a premalignant significance for EGGCTs as well.

## Case presentation

A young 30-year-old South Asian male, a carpenter by occupation with no specific medical history, presented with gradually progressive breathlessness, which increased on leaning forward or lying flat and was relieved by turning to either side. He also had gradually worsening facial puffiness and swelling of the upper limbs, as well as progressive difficulty in swallowing both solids and liquids over the last month. Physical examination showed facial edema, with dilated veins over the chest wall, upper back, and upper arms, and a positive Pemberton sign, suggestive of superior vena cava obstruction. On cardiac auscultation, he had a harsh ejection systolic murmur over the aortic area. An abdominal examination showed no free fluid and normal testes.

Blood investigations (Table 1) showed elevated human chorionic gonadotropin β-subunit and lactate dehydrogenase with normal alpha fetoprotein. Also identified was a chronic hepatitis B infection with a quantitative DNA titer less than 2000 copies/mL. Liver function tests were normal.

**Table 1:** Initial laboratory evaluation of the patient (reference ranges included with the parameter).

Parameter	Patient value	Parameter	Patient value	Parameter	Patient value
Hemoglobin (g/dL, 13-17)	11.4	Aspartate aminotransferase (IU/L, <40)	36	Hepatitis B surface antigen	Positive
Total white blood cell count (×10^3^ cells/μL, 4-11)	5.16	Alanine aminotransferase (IU/L, <40)	25	Hepatitis B envelope antigen	Negative
Platelets (×10^3^ cells/μL, 150-450)	238	Alkaline phosphatase (IU/L, 32-120)	248	Hepatitis B virus total DNA (copies/mL)	<2000
Blood urea nitrogen (mg/dL, <20)	21	Total bilirubin (mg/dL, <1.2)	0.74	Lactate dehydrogenase (U/L, 135-225)	589
Creatinine (mg/dL, 0.7-1.2)	1.0	Albumin (g/dL, 3.5-5.2)	4.3	Alpha fetoprotein (ng/mL, <7)	2.190
Thyroid-stimulating hormone (μIU/mL, 0.270-4.200)	2.600	International normalized ratio (0.9-1.1)	1.01	Human chorionic gonadotropin, β-subunit (mIU/mL, <2 in males)	50.32

**Figure 1:**
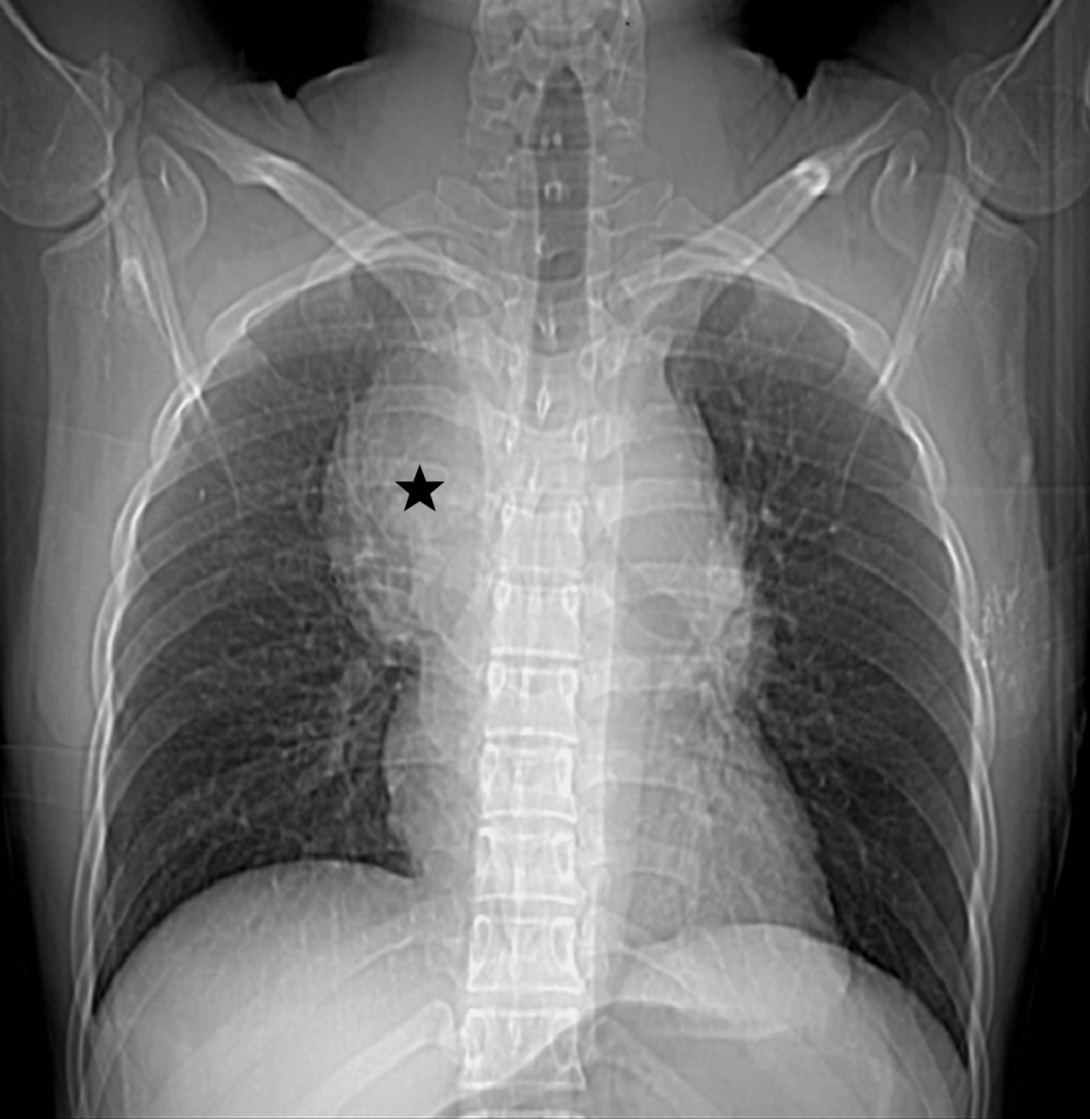
Chest X-ray showing mediastinal widening due to a mass (black star).

A chest X-ray showed an upper mediastinal mass (Figure 1).

**Figure 2:**
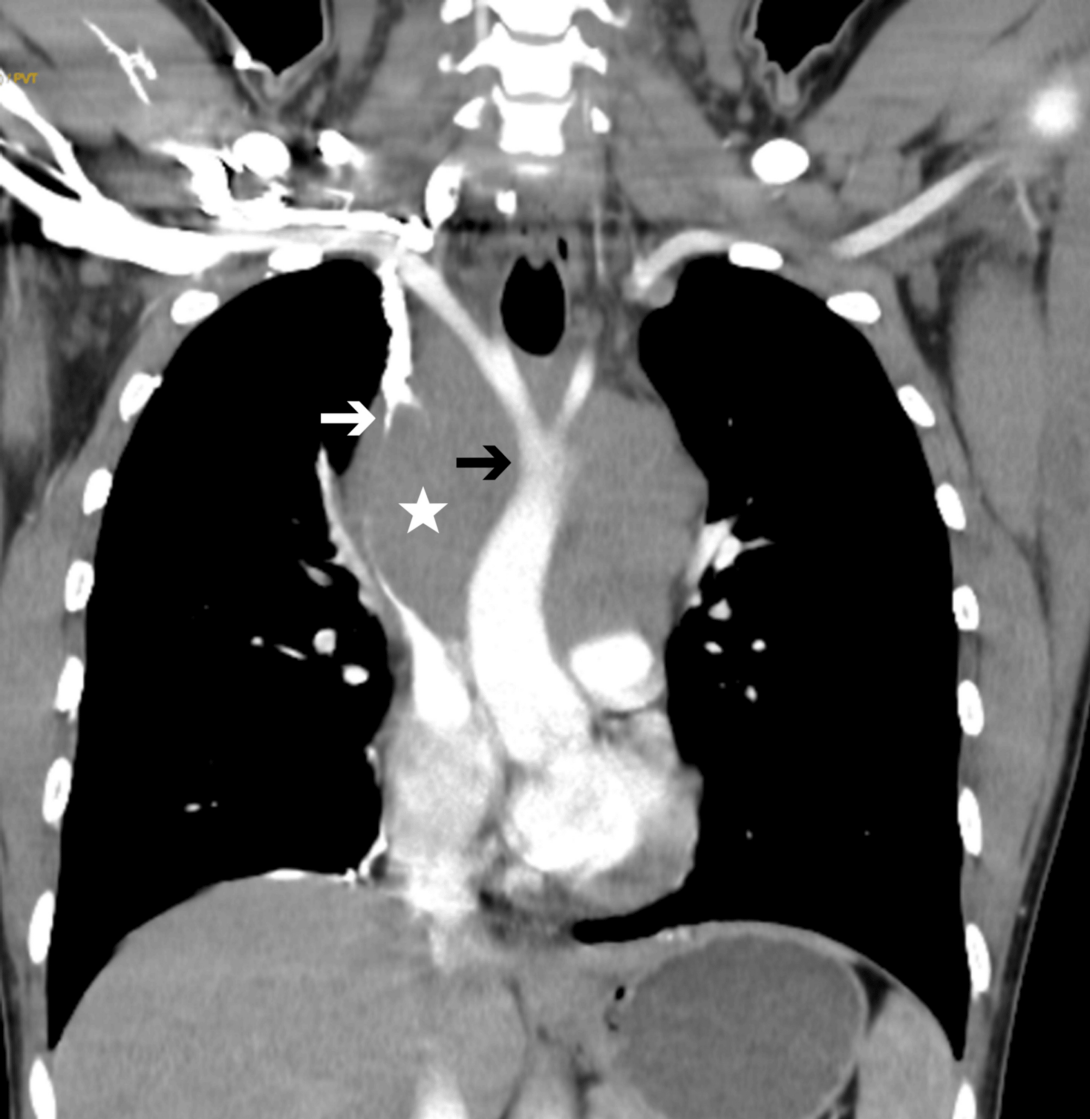
Thorax CT in coronal view showing a large superior mediastinal mass (white star), completely encasing the aorta (black arrow) and causing superior vena cava obstruction (white arrow).

Transthoracic 2D echocardiography (2D-echo) was suggestive of a large extrinsic compression of the arch of the aorta. Computed tomography (CT) of the chest with contrast enhancement showed an 8.6 × 11.3 × 10.9 cm well-defined lesion in the anterior mediastinum with areas of necrosis, with compression of the superior vena cava and right brachiocephalic vein as well as multiple venous collaterals in the anterior chest wall draining into the internal mammary and paravertebral veins (Figure 2).

Whole body positron emission tomography-computed tomography (PET-CT) confirmed a 9.4 × 12.4 × 11.3 cm heterogeneously enhancing fluorodeoxyglucose (FDG)-avid, lobulated soft-tissue mass (maximum standardized uptake value (SUVmax) = 11.8) in the anterior mediastinum with thrombosis of parts of the azygos and right subclavian veins and no other FDG-avid lesions in the body.

**Figure 3:**
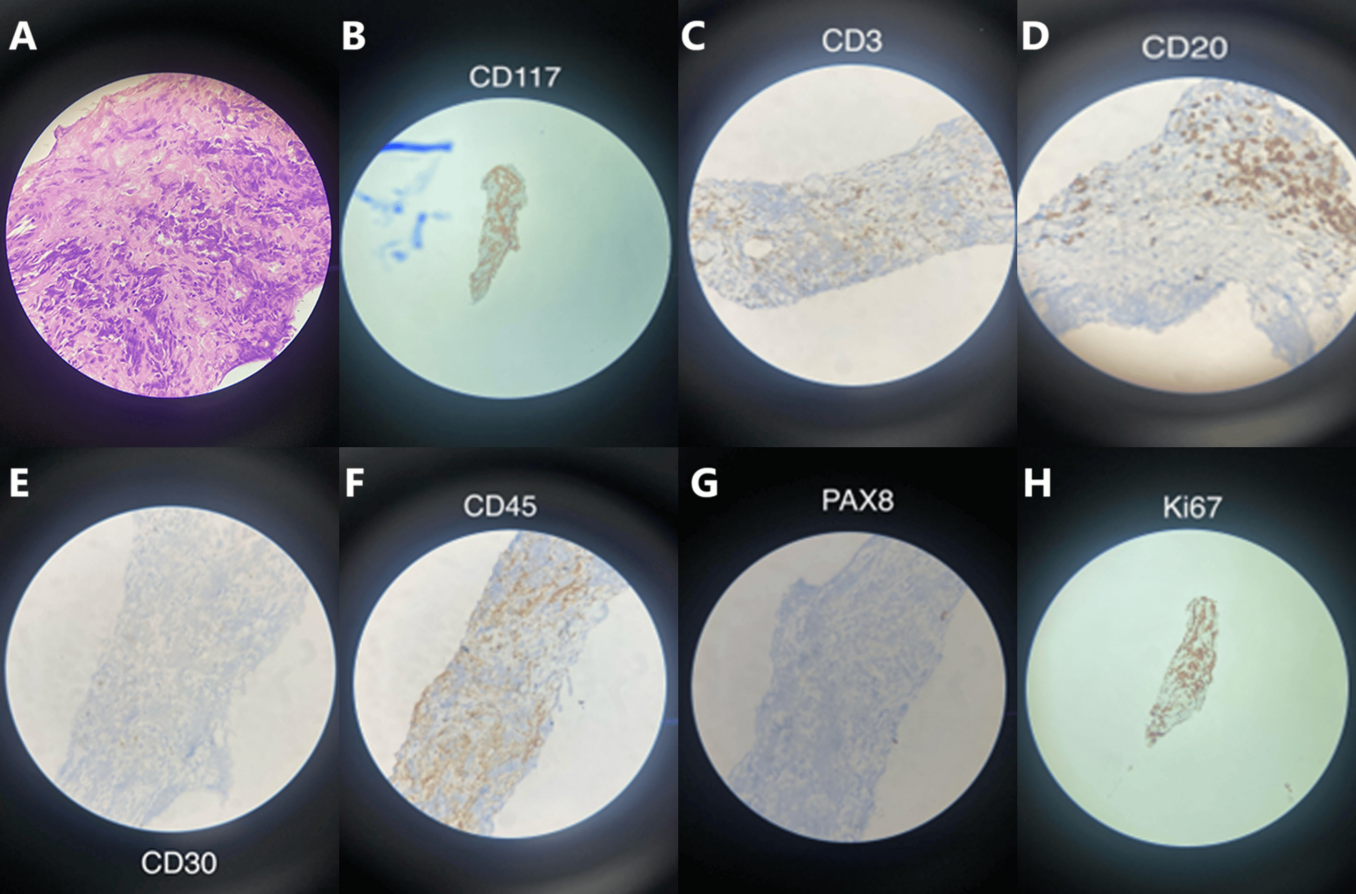
Histopathological (100x magnification) and immunohistochemical (scanning magnification) examination of tissue taken from the mediastinal mass. Pane A: Hematoxylin and eosin (H&E) stain at 100x magnification showing sheets of glycogen-rich tumor cells with fibrous septa and intercellular septa. Pane B: Biopsy stains positive for CD117. Panes C-G: Biopsy stains negative for CD3, CD20, CD30, CD45, and PAX8. Pane H: Ki67 labeling index of the tumor showing 60% positivity.

**Figure 4:**
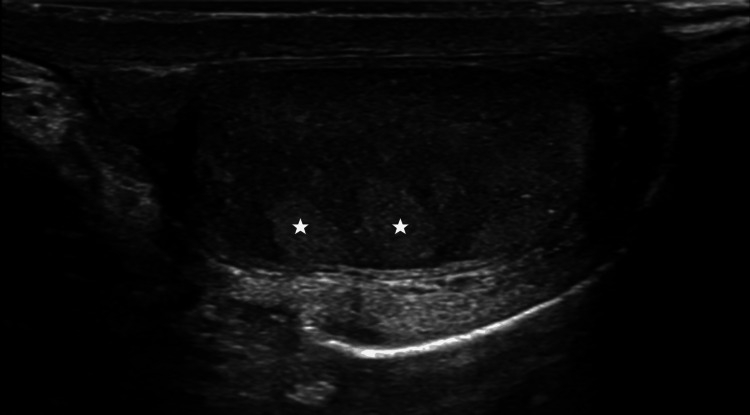
Ultrasonography of the testis showing multiple non-shadowing calcific hyperechogenic foci of varying sizes (of which two have been marked with white stars), suggestive of testicular microlithiasis.

Ultrasonography-guided biopsy of the lesion was performed, and histopathological and immunohistochemical examinations were done (Figure 3). Tissue examination was suggestive of a seminoma and stained positive for CD117 and negative for CD3, CD20, CD45, CD15, CD30, and PAX8. The Ki-67 labeling index was 60%. Ultrasound screening of the testes revealed left testicular microlithiasis with no evidence of a primary gonadal lesion (Figure 4).

The patient was initiated on a cisplatin-based chemotherapy regimen and, after an induction phase of five days, was planned for further cycles. At the two-month review appointment, the patient is symptomatically doing better with partial resolution of the symptoms.

## Discussion

EGGCTs are usually slow to grow, unlikely to metastasize, and often found on incidental chest imaging. They are confirmed by core needle biopsy of the lesion, supported by serum tumor markers and immunohistochemistry. It is important to note the location of the mass as well as the displacement of structures in the mediastinum. When symptomatic, they present with features related to mass effect, sometimes causing cardiac or respiratory symptoms; they may rarely cause tumor thrombosis or distant metastasis [5]. Histologically, mediastinal seminomas are identical to testicular seminomas, characterized by clear glycogen-rich cytoplasm, with distinct cell borders and round cells growing in nesting patterns with fibrous septa [6]. Malignant GCTs secrete alpha-fetoprotein (AFP), beta human chorionic gonadotropin (β-HCG), and embryonal carcinoma components. Pure seminomas do not secrete AFP, and this can be used to distinguish them from mixed GCTs. β-HCG levels are elevated in one-third of the mediastinal seminomas, but levels greater than 1000 mIU/mL should raise suspicion of mixed GCT [7]. PET-CT can be used to detect response to treatment, pick up nodal or distant metastasis, as well as exclude a gonadal primary lesion. Treatment is always chemotherapy and radiation, irrespective of tumor size. Surgical treatment is reserved for failure of response or for residual disease after chemotherapy and radiation [6,7].

While TM is a known pre-malignant condition for testicular germ cell malignancy, no such association exists for EGGCTs, with reports of their coexistence being sparse in the literature. An initial theory posited that the extragonadal sites represented metastases from an occult primary, but a case series of 20 patients with extragonadal mediastinal GCTs found only one case of a testicular primary and one patient with a testicular scar [8]. Another hypothesis suggests that EGGCTs represent the metastases of primary gonadal GCTs, with the primary having regressed (“burned out”) at the time of presentation [9]. Histologic studies of the testes of patients with extragonadal tumors have shown that some had testicular scars in the form of fibrous tissue and microlithiasis that may represent these burned-out primaries [6]. The increase in cumulative risk of metachronous testicular cancer 10 years after the diagnosis of the EGGCT [10] represents a need to determine any possible link between TM and EGGCTs to follow up these patients and screen periodically for the development of new malignancy at high-risk sites.

## Conclusions

This is an uncommon presentation of a primary extragonadal germ cell malignancy concomitant with TM. TM may be a pre-malignant condition for extragonadal malignancy; it may also represent a burned-out primary or a high-risk site for future malignancy. In the context of an existing EGGCT, patients with this finding require close surveillance to identify metachronous testicular malignancy early.
